# Conformational exchange of aromatic side chains by ^1^H CPMG relaxation dispersion

**DOI:** 10.1007/s10858-018-0210-5

**Published:** 2018-09-18

**Authors:** Heiner N. Raum, Matthias Dreydoppel, Ulrich Weininger

**Affiliations:** Institute of Physics, Biophysics, Martin-Luther-University Halle-Wittenberg, 06120 Halle (Saale), Germany

**Keywords:** Conformational exchange, Protein dynamics, Aromatic side chains, Strong couplings

## Abstract

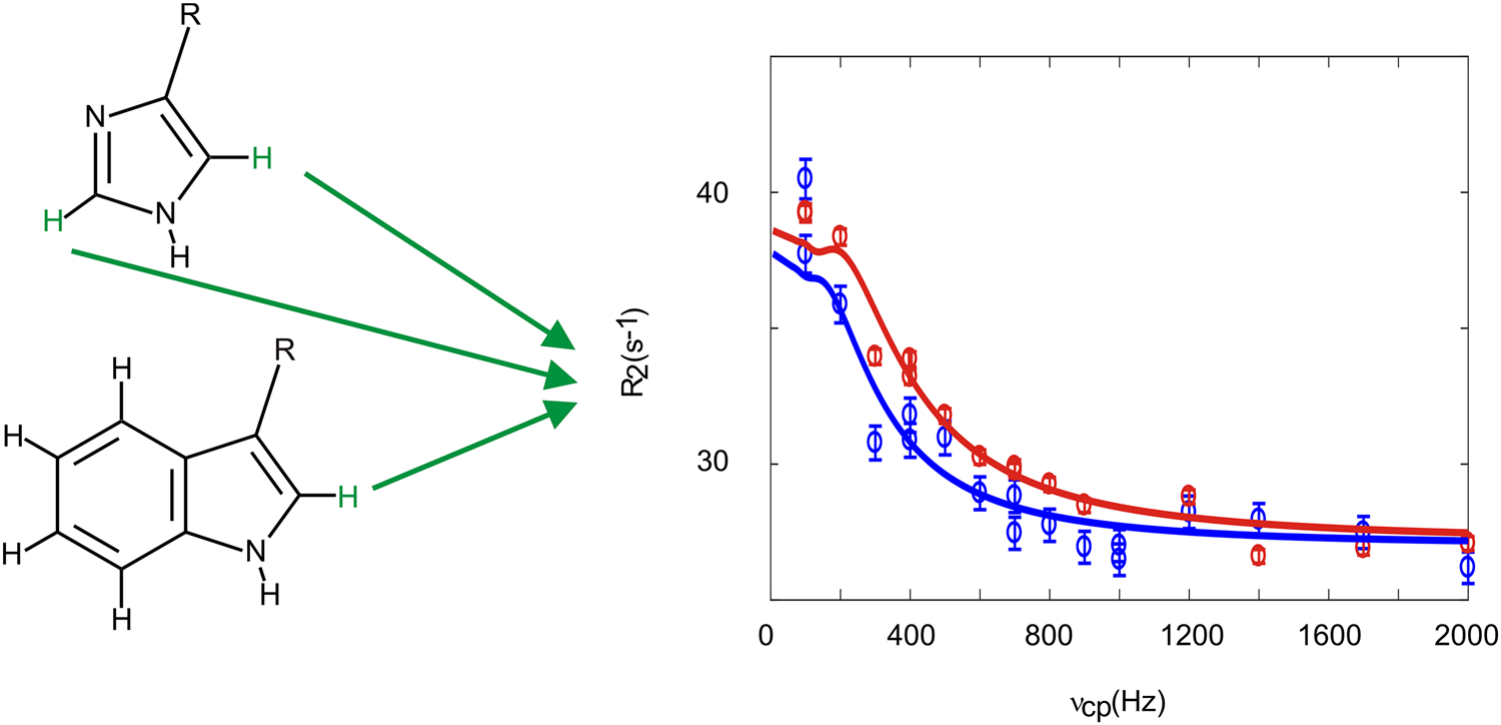

## Introduction

Proteins are dynamic entities that continuously undergo dynamic processes on various time scales. Especially conformational transitions on the millisecond time scale are often linked to biological function (Mittermaier and Kay 2009) and transiently populated high-energy states play important roles in enzyme catalysis (Boehr et al. 2006; Cole and Loria 2002; Eisenmesser et al. 2002) or ligand binding (Demers and Mittermaier 2009; Malmendal et al. 1999). Such transitions between different conformations generally lead to a modulation of NMR parameters as the chemical shift (Gutowsky and Saika 1953) or residual dipolar couplings (Igumenova et al. 2007; Vallurupalli et al. 2007), resulting in exchange contributions to transverse relaxation rate constants. This can be probed by NMR relaxation dispersion methods from which one can gain unique information on the structures, thermodynamics and dynamics of the underlying processes (Palmer 2004; Palmer et al. 2001). To date, Carr–Purcell–Meiboom–Gill (CPMG) experiments (Carr and Purcell 1954; Meiboom and Gill 1958), that cover the millisecond time scale, have been designed for amide ^15^N (Loria et al. 1999a, b) and ^1^H (Ishima and Torchia 2003), backbone CO (Lundström et al. 2008) and Cα (Lundström et al. 2009a), aliphatic side chain Cβ (Lundström et al. 2009b) and ^1^H (Hansen et al. 2012), side chain CO (Hansen and Kay 2011), methyl groups (Baldwin et al. 2010; Mulder et al. 2002; Otten et al. 2010; Weininger et al. 2012b) and aromatic side chain ^13^C (Weininger et al. 2012c).

Aromatic side chains are bulky and responsible for a significant proportion of the protein hydrophobic core. They typically form pairs or clusters where they make specific aromatic–aromatic interactions (Burley and Petsko 1985, 1989). They are prevalent in protein binding interfaces (Trp is four times enriched in binding sites, relative to their natural occurrence, Tyr > 2 times, the only other amino acid > 2 is Arg), where they contribute significantly to the binding free energy (Birtalan et al. 2010; Bogan and Thorn 1998; Lo Conte et al. 1999). His and Tyr (18% and 6% of all catalytic residues) also play prominent roles in enzyme catalysis (Bartlett et al. 2002). All this makes aromatic side chains interesting and useful probes for studying protein dynamics. Even more, Phe and Tyr undergo frequent 180° rotations of the χ^2^ angle (‘ring flips’) and thereby provide unique information of transient ‘breathing’ processes of proteins (Li et al. 1999; Wagner 1980; Wagner et al. 1976). His can exist in three different states, one protonated and two different neutral tautomeric forms. Transient changes between these states affect hydrogen bonding patterns around the histidine. Thus, it is of great interest to monitor the dynamics of aromatic residues. With easy and robust labeling protocols to achieve site-selective ^13^C labeling (Lundström et al. 2007; Teilum et al. 2006; Weininger 2017) studies of dynamics on aromatic side chains have come into focus. Improved methods of obtaining relaxation rate constants have been developed (Weininger et al. 2012a) and the first studies of order parameters have been reported (Boyer and Lee 2008; Kasinath et al. 2013, 2015). ^13^C relaxation dispersion experiments for the study of dynamics on the ms (Weininger et al. 2012c) and µs (Weininger et al. 2014a) time scale have been developed and applied on the characterization of ring flips (Weininger et al. 2013, 2014b) and transient histidine tautomerization (Weininger et al. 2017). Furthermore, residual dipolar couplings have been obtained (Sathyamoorthy et al. 2013).

So far, all this new studies of aromatic side chain dynamics are based on ^13^C. Here we investigate the possibility of ^1^H CPMG relaxation dispersion experiments in aromatic side chains, as a complementary method. Some processes will more reflect in ^1^H others in ^13^C CPMG relaxation dispersion profiles. ^1^H CPMG relaxation dispersion experiments can be affected by ^3^J ^1^H–^1^H couplings and ^13^C–^13^C strong couplings. Artifact-free relaxation dispersion profiles can be obtained in uniformly ^1^H and ^13^C labeled samples in case of His δ2, His ε1 and Trp δ1, since ^3^J ^1^H–^1^H couplings are sufficiently small and ^13^C–^13^C strong couplings do not occur in these positions. Other positions require site-selective ^13^C labeling in case of ^13^C–^13^C strong couplings and site-selective deuteration in all cases. ^1^H CPMG relaxation dispersion experiments were applied and verified on unfolding of CspB (Weininger et al. 2012c; Zeeb and Balbach 2005) for His δ2, His ε1 and Trp δ1, therefore providing a complementary approach to ^13^C CPMG relaxation dispersion experiments.

## Materials and methods

### Protein samples

Uniformly ^15^N^13^C labeled ubiquitin at pH 6.5 was purchased from ASLA. Uniformly ^15^N^13^C labeled and site-selective ^13^C labeled, using 1-^13^C and 2-^13^C glucose (Lundström et al. 2007; Teilum et al. 2006), GB1 (QDD variant) at pH 7.0 in water was expressed and purified as described in Lindman et al. (2006). Site-selective ^13^C labeled, using 1-^13^C glucose, SlyD at pH 7.4 in 20 mM HEPES was purified and described as in Löw et al. (2010). Uniformly ^13^C labeled CspB at pH 7.0 in 10 mM HEPES was expressed and purified as described in Schindler et al. (1995). Uniformly ^13^C labeled *human* carbonic anhydrase II, at pH 7.7 in 50 mM TRIS/HCl was expressed and purified as described in Michalczyk et al. (2015).

### NMR spectroscopy

All experiments were acquired on a Bruker Avance III spectrometer at a static magnetic field of 14.1 T and 298 K. Additionally, relaxation dispersion experiments on CspB were recorded on Bruker Avance III spectrometer at a static magnetic field of 18.8 T. Samples contained 1–3 mM protein (except carbonic anhydrase, which was below 100 µM) and 10% (v/v) D_2_O. ^1^H CPMG relaxation dispersion experiments were performed with refocusing frequencies between 100 and 1000 Hz (2000 Hz in case of CspB) and B1 field strengths for the CPMG pulses between 13 and 15 kHz. Spectra were processed with NMRPipe (Delaglio et al. 1995) and analyzed with PINT (Ahlner et al. 2013).

### Data analysis

CPMG relaxation dispersion experiments were fitted globally to the Carver–Richards equation (Carver and Richards 1972; Davis et al. 1994). Data modeling utilized the Levenberg–Marquardt (Press et al. 2002) nonlinear least-squares optimization algorithm implemented in MATLAB. Derived Δδ values were compared with ^1^H shift differences between native and extrapolated unfolded signals from a NMR urea titration (Weininger et al. 2012c).

### Density matrix calculations

The evolution of the spin density operator in Liouville space during the ^1^H CPMG experiment was simulated by way of the homogeneous master equation (Jeener 1982; Levitt and Dibari 1992) as implemented in Qsim (Helgstrand and Allard 2004). Cross relaxation effects were included. The simulations covered the full CPMG relaxation period. Simulations were performed for 3- or 4-spin systems for the directly coupled ^1^H-^13^C spin pair of interest to one ^1^H, two ^1^H, or one ^1^H bound to ^13^C, for ^3^J ^1^H–^1^H coupling constants of 2 Hz or 8 Hz. The ^1^H carrier was set 1000 Hz away from the proton of interest. The loss of magnetization was used to calculate an artificial *R*_ex_ contribution.

## Results and discussion

### Couplings in aromatic side chains

**Fig. 1:**
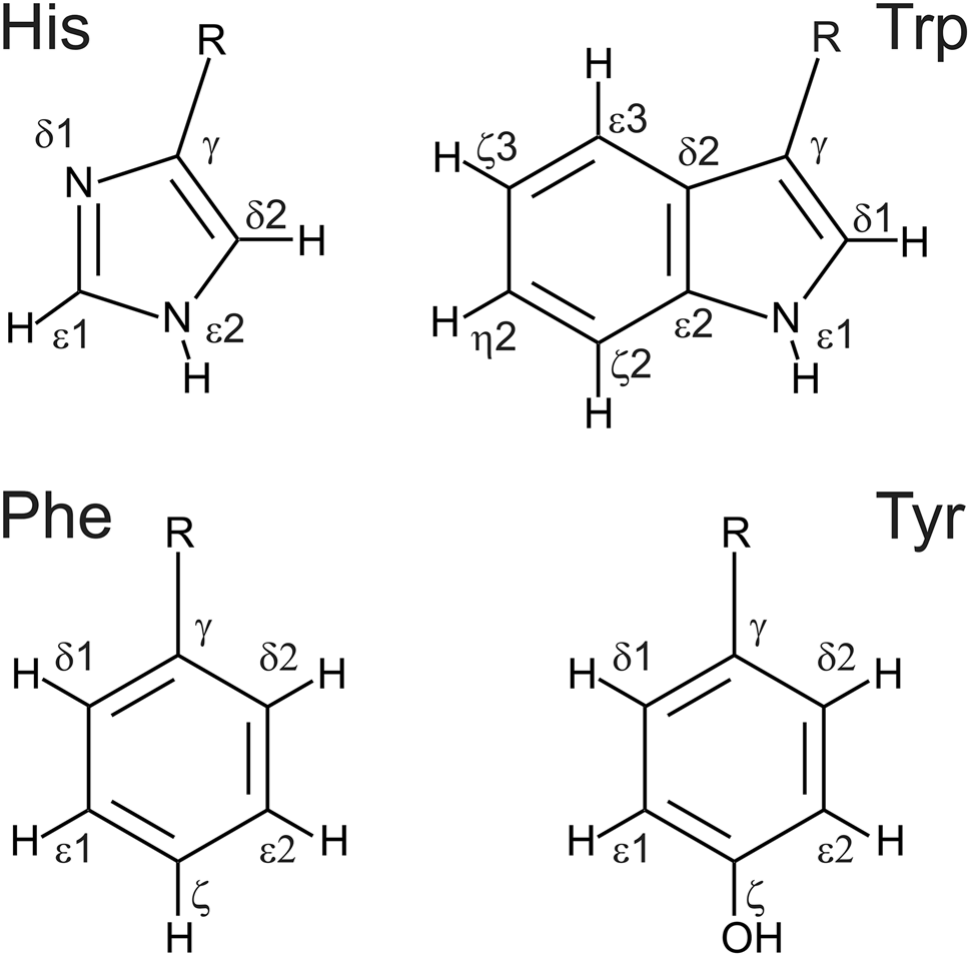
Aromatic side chains (His, Trp, Phe and Tyr) with different positions labeled. δ1, δ2 and ε1, ε2 in Phe and Tyr are usually averaged to δ* and ε* because of fast ring flips. His is shown in its most stable neutral tautomeric form

Acquiring artifact-free ^1^H relaxation dispersion profiles in aromatic side chains (Fig. 1) can potentially be prevented by different J-couplings within the aromatic ring. First and foremost by ^3^J ^1^H–^1^H couplings of a certain size. ^3^J ^1^H–^1^H couplings in Phe, Tyr and the six-ring moiety of Trp are around 8 Hz as evaluated from ^1^H spectra of the free amino acids. In contrast, no J-couplings between carbon bound protons and nitrogen bound protons in His (δ2 and ε1) and the five-ring moiety of Trp (δ1) could be detected. It was therefore concluded that these couplings are 2 Hz or smaller. Additionally, ^1^H–^1^H or ^13^C–^13^C strong couplings (chemical shift difference of the coupling nuclei is not sufficiently larger than the coupling constant) could affect the relaxation dispersion profiles. Strong couplings can lead to severe additional losses of magnetization, at high or low CPMG refocusing frequencies (Weininger et al. 2013). ^1^H–^1^H strong coupling can be expected in Phe, Tyr and the six-ring moiety of Trp, but not in His (δ2 and ε1) and the five-ring moiety of Trp (δ1), because the chemical shift of the nitrogen bound proton is too different (Ulrich et al. 2008). Additionally, the underlying ^3^J coupling constant of 2 Hz or below is too small to matter (Weininger et al. 2013). ^13^C–^13^C strong coupling can be expected in Phe, Tyr (Cγ–Cδ) and the six-ring moiety of Trp, but again not in His (δ2 and ε1) and the five-ring moiety of Trp (δ1), as evaluated from possible chemical shifts (Ulrich et al. 2008). In Trp, possible strong couplings can directly be evaluated from the spectra. In Phe and Tyr, were signals are most often averaged because of fast ring flips (Wagner et al. 1976), this is often not possible.

### Acquiring ^1^H CPMG relaxation dispersion profiles

**Fig. 2:**
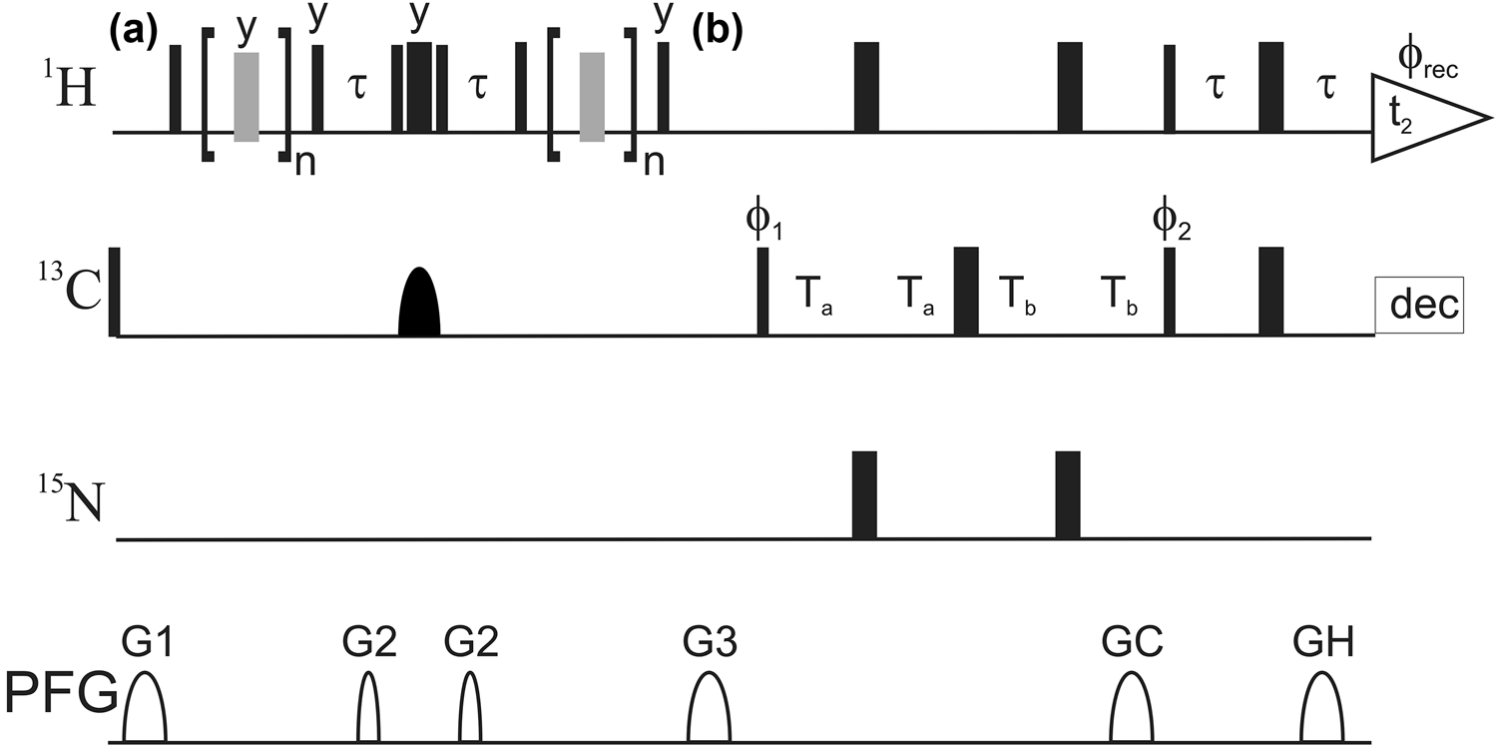
Pulse sequence of the ^1^H CPMG relaxation dispersion experiment for measuring conformational exchange of aromatic side chains. All pulses are applied along the *x*-axis unless otherwise indicated. Narrow (wide) solid bars indicate rectangular high-power 90° (180°) pulses. Wide grey bars indicate 180° pulses in the CPMG elements, which have attenuated power. The wide semi-ellipse on ^13^C represents a REBURP (Geen and Freeman 1991) pulse with a bandwidth of 40 ppm. All proton pulses after (*a*) are applied on resonant on the aromatics, after (*b*) on-resonant on water. The delay τ can be set to 1.6 ms (Phe and Tyr), 1.35 ms (all aromatics) or 1.25 ms (His). T_a_ and T_b_ are 1.1 ms and 1.1 ms + t_1_/2 for non constant-time detection and 4.464 ms − t_1_/4 and 4.464 ms + t_1_/4 for constant-time detection, respectively. The pulses flanking the CPMG blocks purge non-refocused magnetization remaining as a consequence of the variation among aromatic sites in the ^1^*J*_HC_ coupling constant (Vallurupalli et al. 2007; Weininger et al. 2012c). The phase cycle is: ϕ_1_ = (x, − x), ϕ_2_ = (x, x, − x, − x), ϕ_rec_ = (x, − x, − x, x). Pulsed field gradients G1–3 are employed to suppress unwanted coherences and artifacts, while GC and GH are encoding and decoding gradients, respectively, for echo/anti-echo coherence selection, obtained by inverting the signs of GH (Kay et al. 1992). For every second t_1_ increment ϕ_1_ and the receiver were incremented. Gradient durations (in ms) and relative power levels (in %) are set to (duration, power level) G1 = (1.0, 13), G2 = (0.5, 10), G3 = (1.0, 90), GC = (1.0, 80), GH = (1.0, − 20.1)

The pulse sequence for measurement of ^1^H CPMG relaxation dispersions in aromatic side chains is shown in Fig. 2. It uses the relaxation compensation approach between proton inphase and proton–carbon antiphase magnetization (Loria et al. 1999a). To account for different ^1^H–^13^C J-couplings in aromatic side chain purging 90° pulses are applied on both sides of the block converting inphase to antiphase magnetization (Vallurupalli et al. 2007; Weininger et al. 2012c). 2D detection can be achieved in a constant-time or non constant-time approach, while water is suppressed by selection gradients (G_C_ and G_H_). Since the TROSY effect for aromatic protons is neglectable, no TROSY selection of the relaxation rate constant was applied (Loria et al. 1999b; Weininger et al. 2012c). Also shaped CPMG pulses on proton are not practical, because of their long duration, which would allow only low refocusing frequencies. In summary ^1^H CPMG relaxation dispersions can be recorded in a simple, robust and straightforward way.

### Simulation of ^3^J ^1^H–^1^H couplings

**Fig. 3:**
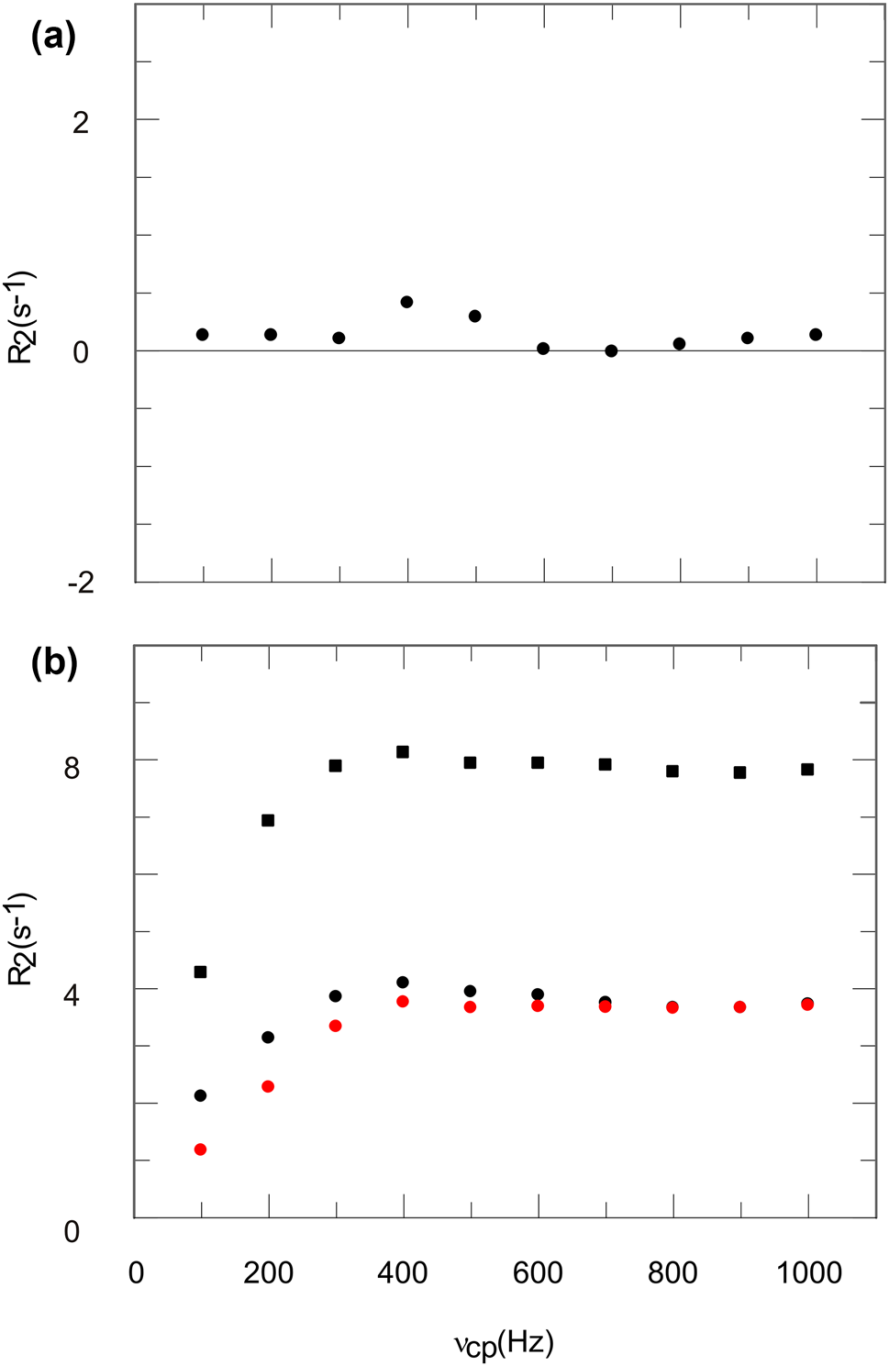
Simulation of additional *R*_2_ contributions in aromatic ^1^H CPMG relaxation dispersion profiles caused by ^3^J ^1^H–^1^H couplings. Simulations were performed with Qsim (Helgstrand and Allard 2004). **a** Assuming a J-coupling of 2 Hz, which is the maximal J-coupling in case of His δ2, His ε1 and Trp δ1 (RMSD 0.12 s^−1^). **b** Assuming a J-coupling of 8 Hz, which is the coupling in Phe, Tyr and the six-ring moiety of Trp. Here, simulations were performed for one coupling ^1^H (black circles), two coupling ^1^H (black squares) or one coupling ^1^H which itself is directly coupling to ^13^C (red circles)

In order to quantify the disturbance of aromatic ^1^H CPMG relaxation dispersion profiles caused by ^3^J ^1^H–^1^H couplings, density operator simulations of the CPMG relaxation period were performed, including cross relaxation effects. For His δ2, His ε1 and Trp δ1 simulations were performed assuming a maximal J-coupling of 2 Hz. The effect of this J-coupling on the relaxation dispersion is minimal (Fig. 3a). Relaxation rate constants are not affected at all for most refocusing frequencies, resulting in a rate constant of zero. Only for refocusing frequencies of 400 Hz and 500 Hz a small increase of 0.5 Hz is observed, which should be well within the experimental noise. Also the simulation is already the worst case scenario, assuming 2 Hz coupling. In Phe, Tyr and the six-ring moiety of Trp, the J-coupling is 8 Hz. Here a clear artificial contribution to the relaxation dispersion profile is observed (Fig. 3b). In case of one coupling proton the artificial dispersion step is 2 s^−1^, for two coupling protons it is 4 s^−1^. In case the coupling proton is connected to ^13^C, the effect is slightly more severe, than for just one coupling proton. In summary the simulations show that ^3^J ^1^H–^1^H couplings should not be a problem for His δ2, His ε1 and Trp δ1, but lead to certain artifacts in other positions.

### Off-resonance effects

**Fig. 4:**
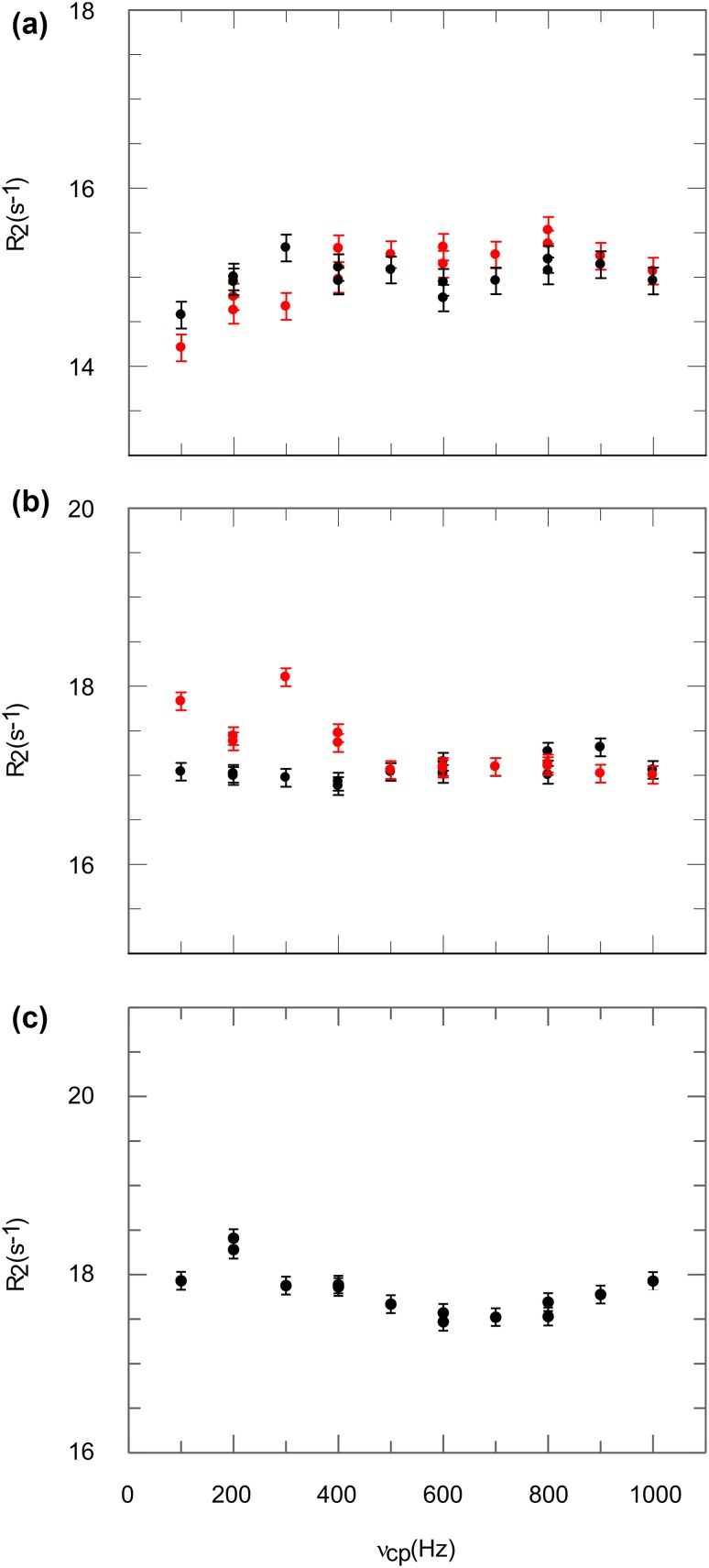
Aromatic ^1^H CPMG relaxation dispersion profiles of ubiquitin H68 δ2 (**a**), H68 ε1 (**b**) and GB1 W43 δ1 (**c**). Black symbols represent a close to on-resonant carrier during the CPMG block, red symbols an off-resonant carrier. RMSD values for on-resonant experiments (black symbols) are 0.18 s^−1^, 0.12 s^−1^, 0.26 s^−1^

In order for the CPMG block to work as intended, it is often crucial that the pulses are applied close to on-resonant. This was investigated on His 68 of ubiquitin. One CPMG relaxation dispersion experiment was performed with the ^1^H carrier during the CPMG block at 7.5 ppm, a second with the carrier at 7.0 ppm. The first experiment is within 150 Hz on-resonant on H68 ε1, the second on H68 δ2. Accordingly the other experiments are performed within 400–500 Hz off-resonant to the respective His position. The on-resonant cases (Fig. 4a, b, black symbols) result in flat dispersion profiles, as one can expect for positions with low ^3^J ^1^H–^1^H couplings in a system with known absence of ms exchange. In contrast already for somewhat off-resonant cases (Fig. 4a, b, red symbols) the relaxation dispersion profile is affected. The severe influence of the ^1^H carrier during the CPMG block is unexpected and not in agreement with the simulation. Although this effect is not understood, artifact-free relaxation dispersion profiles can be acquired if CPMG pulses are applied close to on-resonant, in our experience within 300 Hz to the signals of interest.

### Artifact-free ^1^H CPMG dispersion profiles of His δ2, His ε1 and Trp δ1

Density operator simulations, as well as considerations of strong couplings, lead to the conclusion, that artifact-free aromatic ^1^H CPMG relaxation dispersion profiles can be obtained for His δ2, His ε1 and Trp δ1. This was validated experimentally by studying two model proteins with known absence of ms dynamics at 25 °C, ubiquitin for His δ2 and ε1 (H68), and GB1 for Trp δ1 (W43). They all show artifact-free flat dispersion profiles, as one should expect for the absence of ms dynamics (Fig. 4, black symbols). Hence, these positions are suitable for aromatic ^1^H CPMG relaxation dispersion experiments in uniformly or site-selectively ^13^C labeled proteins.

### Effect of couplings and strong couplings in aromatic six-ring moieties

**Fig. 5:**
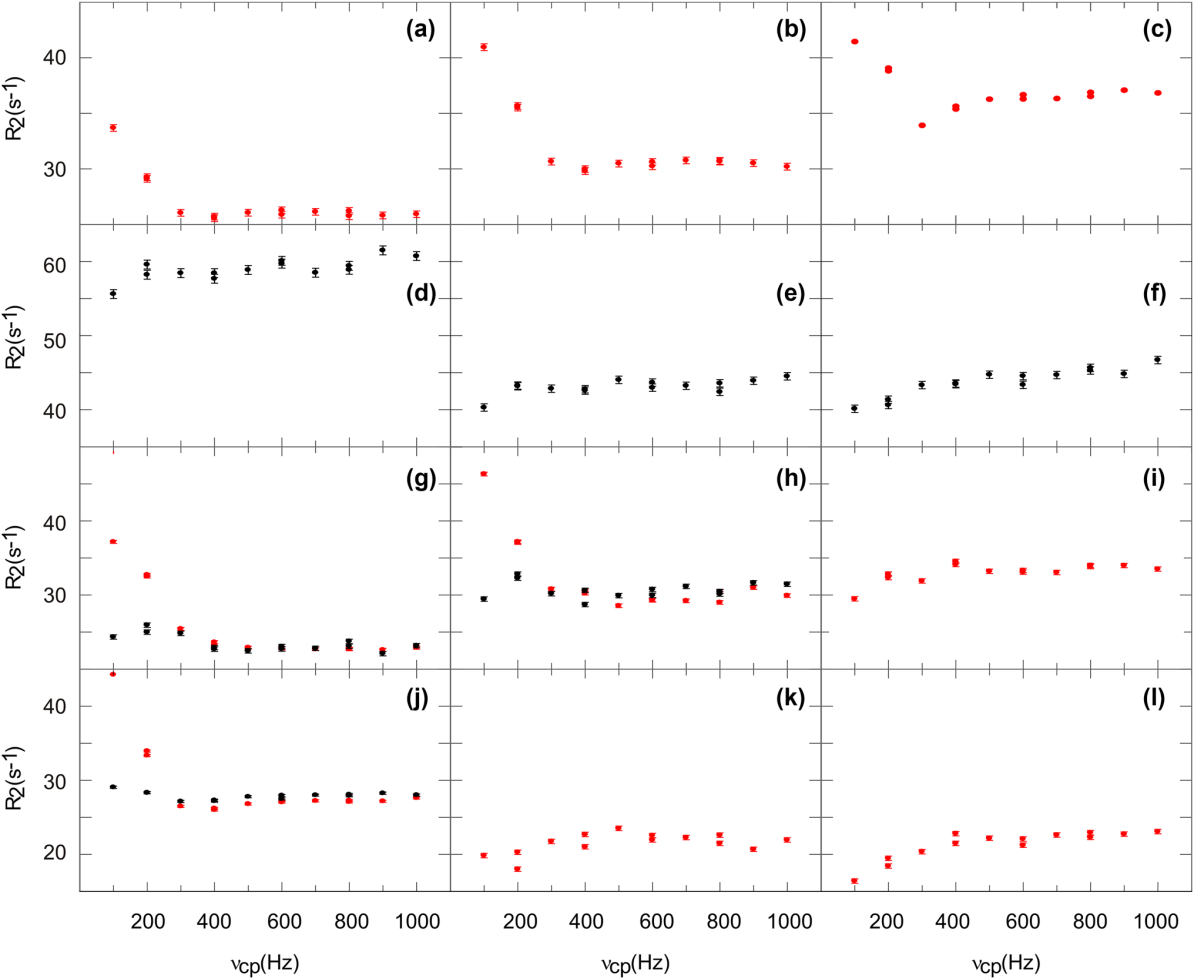
Aromatic ^1^H CPMG relaxation dispersion profiles of ubiquitin (**a**–**c**) F4 δ* (**a**), F4 ε* (**b**), Y59 δ* (**c**), tSlyD (**d**–**f**) F79 δ* (**d**), F91 δ* (**e**), F117 δ* (**f**), GB1 (**g**–**l**) W43 ε3 (**g**), W43 ζ3 (**h**), W43 η2 (**i**), F52 δ* (**j**), Y33 δ* (**k**) and Y33 ε* (**l**). Red symbols represent measurements on uniformly ^13^C labeled samples, black symbols measurements on site-selective ^13^C labeled samples

Density operator simulations of aromatic six-ring moieties (^3^J ^1^H–^1^H of 7–8 Hz) show an artificial increase of *R*_2_ in the dispersion profiles with increasing refocusing frequencies caused by the ^3^J ^1^H–^1^H coupling (Fig. 3b). Experimental relaxation dispersion profiles of uniformly ^13^C labeled ubiquitin also show this behavior for refocusing frequencies of 300–1000 Hz, but also display additional very high *R*_2_ values for refocusing frequencies of 100 and 200 Hz (Fig. 5a–c). In contrast, relaxation dispersion profiles of site-selectively ^13^C labeled tSlyD only showed the profile expected from the simulation (Fig. 5d–f). The low frequency artifact in ubiquitin can be addressed to ^13^C–^13^C strong couplings. Strong couplings of the nuclei directly coupled to the nuclei investigated by CPMG relaxation experiments can have dramatic effects on the dispersion profiles (Weininger et al. 2013). Phe (Fig. 5a, b) are likely to display ^13^C–^13^C strong couplings, while Tyr δ (Fig. 5c) can display ^13^C–^13^C strong couplings to Tyr γ (Ulrich et al. 2008). In order to elaborate this further, relaxation dispersion profiles were acquired on uniformly and site-selectively ^13^C labeled GB1 (Fig. 5g–l). Trp ε3 and ζ3 (Fig. 5g, h) are strongly ^13^C–^13^C coupled without a doubt, as directly seen from the spectra. They show extremely high *R*_2_ values at low refocusing frequencies in case of uniformly, but not for site-selectively ^13^C labeling. Trp η2 (Fig. 5i) is not strongly ^13^C–^13^C coupled, as seen from the spectra, and shows the expected profile even with uniform ^13^C labeling. Additionally, for Phe δ (Fig. 5j) the low frequency artifact can be observed for uniform, but not for site-selective ^13^C labeling and Tyr does also show the expected profiles independent of the labeling. In summary, we experimentally found profiles similar to the simulated ones, that are caused by a sizable ^3^J ^1^H–^1^H coupling. *R*_2_ values increase with higher refocusing frequencies. Additionally, ^13^C–^13^C strong couplings cause very high *R*_2_ values at low refocusing frequencies. ^1^H–^1^H strong couplings in tSlyD F79 (Weininger et al. 2013; Fig. 5d) only cause a constant shift of *R*_2_ values independent of the refocusing frequency.

### Unfolding of CspB by ^1^H CPMG relaxation dispersion

**Fig. 6:**
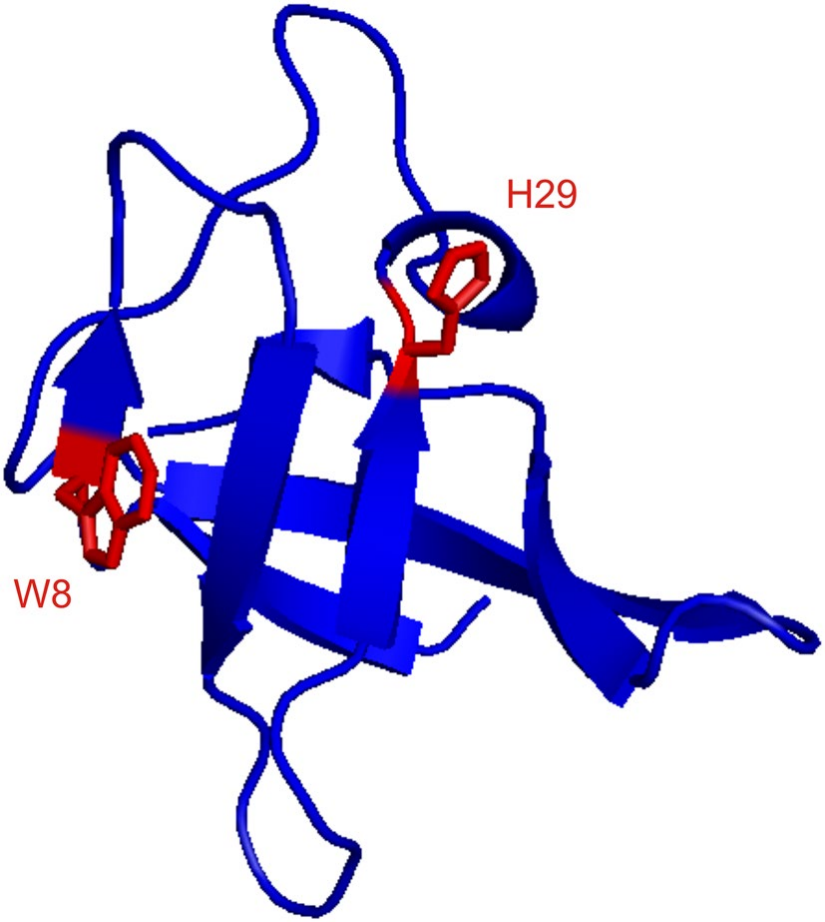
Ribbon representation of CspB using 1csp.pdb. The ribbon is colored in blue, side chains of W8 and are shown as sticks, labeled and colored in red. The figure was generated using PyMOL (Schrodinger 2010)

**Fig. 7:**
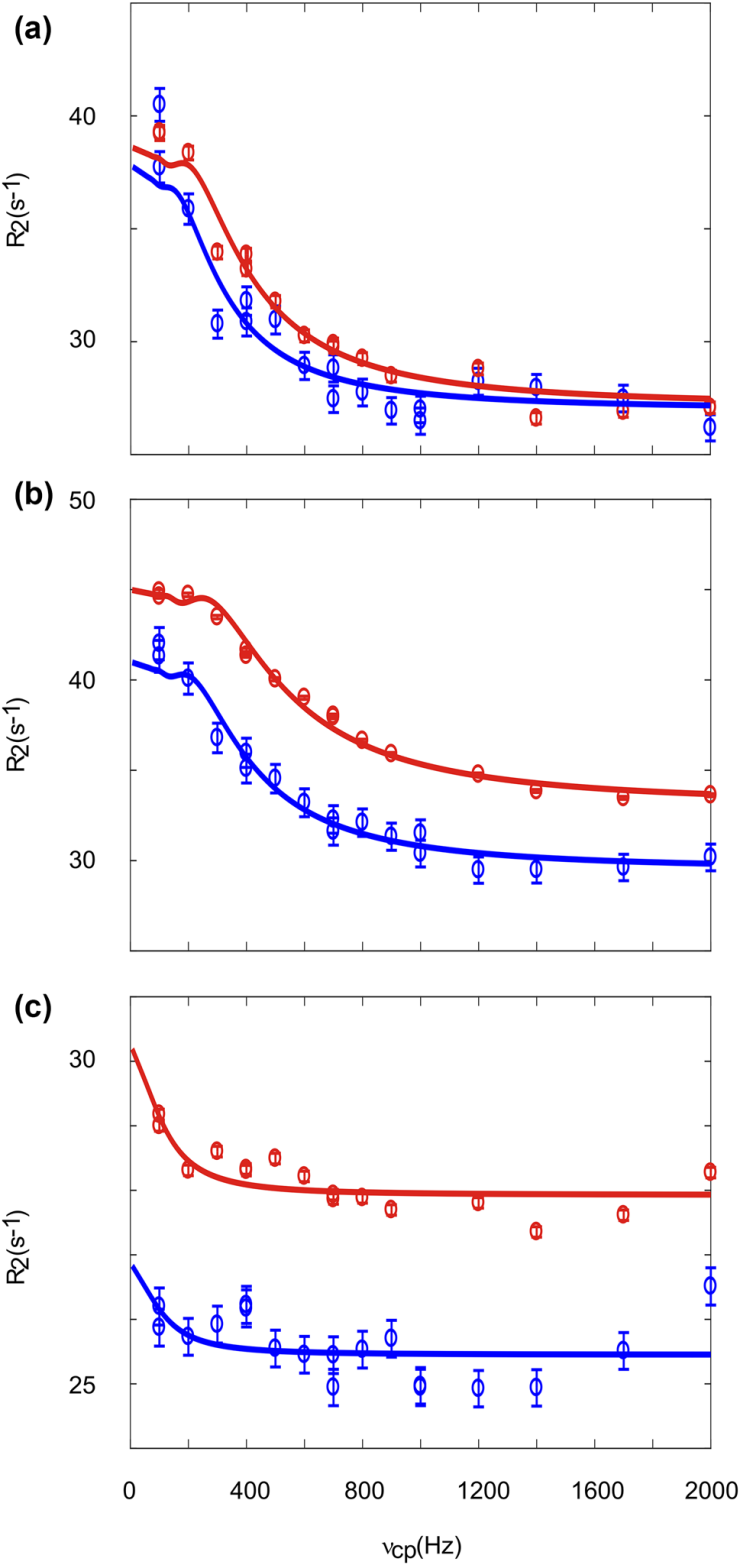
Aromatic ^1^H CPMG relaxation dispersion profiles acquired on a 1.8 mM sample of CspB in 10 mM HEPES pH 7.0 at 25 °C and static magnetic field strengths of 14.1 T (blue) and 18.8 T (red). Data are shown for H29 δ2 (**a**), H29 ε1 (**b**) and W8 δ1 (**c**). Solid lines represent the global fit to the experimental data

**Fig. 8:**
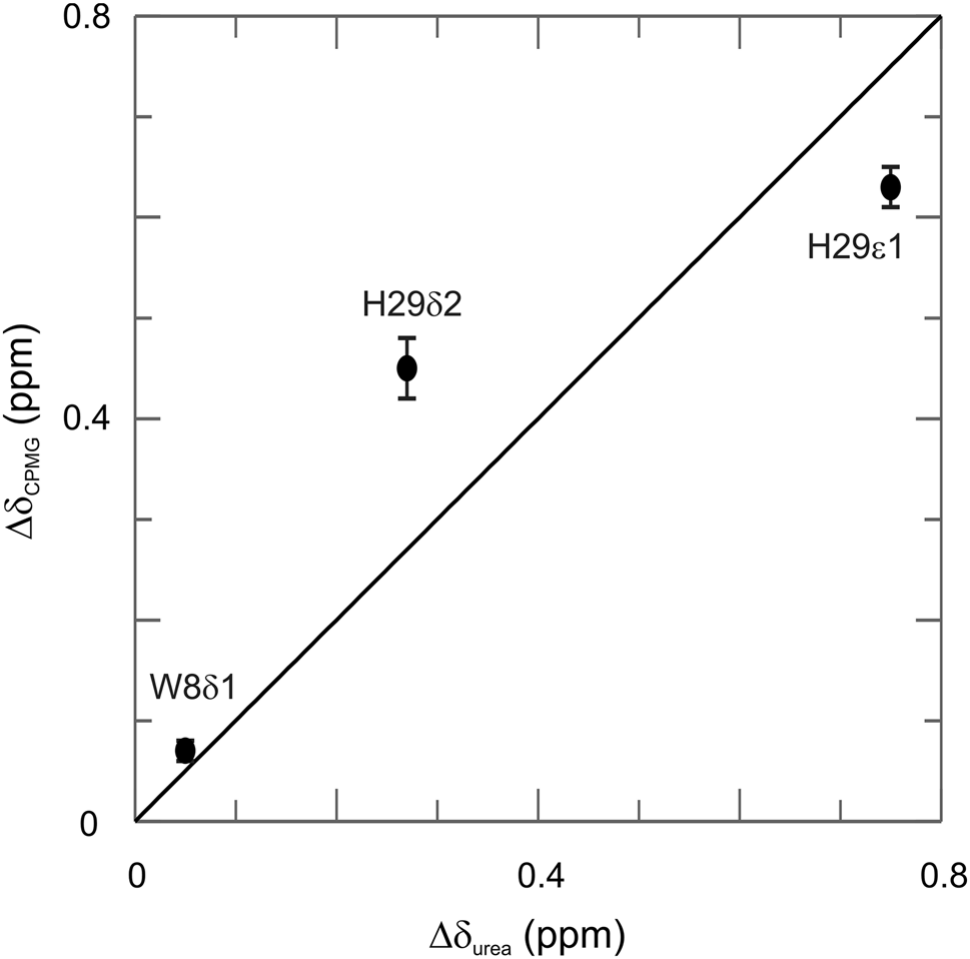
Correlation of ^1^H chemical shift differences between the folded and unfolded states of CspB derived from CPMG relaxation dispersion experiments under native conditions and measured directly from ^1^H–^13^C HSQC spectra in an urea titration experiment. The solid black line represents the ideal correlation

Aromatic ^1^H CPMG relaxation dispersion experiments were applied on CspB from *Bacillus subtilis* (Fig. 6). CspB is folding/unfolding on the ms time scale and has been used as a model system for CPMG relaxation dispersion experiments (Weininger et al. 2012c; Zeeb and Balbach 2005). It contains one Trp and one His, that allow artifact-free ^1^H CPMG relaxation dispersion experiments on H29δ2, H29ε1 and W8δ1. The corresponding dispersion were fitted globally to a two-state exchange model with *k*_ex_ = 690 ± 65 s^−1^ and *p*_u_ = 1.8 ± 0.1% (Fig. 7). The resulting rate constant of unfolding, which is more or less independent from buffer conditions, is *k*_u_ = 12 ± 1 s^−1^. This is in excellent agreement with *k*_ex_ = 528 ± 52 s^−1^, *p*_u_ = 2.3 ± 0.2% and *k*_u_ = 12 ± 3 s^−1^ derived from aromatic ^13^C CPMG relaxation dispersion experiments under the same conditions (Weininger et al. 2012c). *k*_ex_ and *p*_u_ are somewhat dependent on the stabilizing effect of salt, resulting in lower *p*_u_ and higher *k*_ex_ (because of an increased *k*_f_). Chemical shift differences derived from the relaxation dispersion experiments match chemical shift differences derived from a urea transition, back extrapolated to 0 M urea (Fig. 8). Taken together, the unfolding of CspB can be described accurately by aromatic ^1^H CPMG relaxation dispersion experiments on Hδ2, Hε1 and Wδ1.

### Outlook: larger systems

We determined ^1^H *R*_2_ values at high CPMG refocusing frequencies of various Hε1 on human carbonic anhydrase II, a 29 kDa enzyme. These rate constants vary between 10 and 50 s^−1^, which are workable values from a background relaxation perspective. Thus such systems can be investigated by aromatic ^1^H CPMG relaxation dispersion experiments, if enough sensitivity is provided.

## Conclusions

We have investigated experimentally and theoretically the possibility of acquiring ^1^H CPMG relaxation dispersion experiments in aromatic side chains complementary to ^13^C CPMG relaxation dispersion experiments.. Positions with high (7–8 Hz) ^3^J ^1^H–^1^H couplings (Fδ, Fε, Fζ, Yδ, Yε, Wε3, Wζ3, Wη2 and Wζ2) do not allow to acquire artifact-free relaxation dispersion profiles in uniformly protonated samples. They can be impacted further by ^13^C–^13^C strong couplings in uniformly ^13^C labeled samples. In contrast, Hδ2, Hε1 and Wδ1 only have small (< 2 Hz) ^3^J ^1^H–^1^H couplings and no ^13^C–^13^C strong couplings, thus, providing artifact-free relaxation dispersion profiles. Therefore, correct parameters of CspB unfolding could be derived by ^1^H CPMG relaxation dispersion experiments on a uniformly ^1^H and ^13^C labeled sample.
